# Estimated incidence of influenza‐associated severe acute respiratory infections in Indonesia, 2013‐2016

**DOI:** 10.1111/irv.12496

**Published:** 2017-12-05

**Authors:** Ni K. Susilarini, Edy Haryanto, Catharina Y. Praptiningsih, Amalya Mangiri, Natalie Kipuw, Irmawati Tarya, Roselinda Rusli, Gestafiana Sumardi, Endang Widuri, Masri M. Sembiring, Widya Noviyanti, Christina Widaningrum, Kathryn E. Lafond, Gina Samaan, Vivi Setiawaty

**Affiliations:** ^1^ Center for Research and Development for Biomedical and Basic Technology of Health NIHRD, Ministry of Health Central Jakarta Indonesia; ^2^ Acute Respiratory Infection Sub Directorate Directorate General of Disease Control and Prevention Ministry of Health Central Jakarta Indonesia; ^3^ US Centers for Disease Control and Prevention Jakarta Indonesia; ^4^ World Health Organization Central Jakarta Indonesia; ^5^ Influenza Division US Centers for Disease Control and Prevention Atlanta GA USA; ^6^ National Centre for Epidemiology and Population Health Australian National University Canberra ACT Australia

**Keywords:** disease burden, Indonesia, influenza, severe acute respiratory infection, surveillance

## Abstract

**Background:**

Indonesia's hospital‐based Severe Acute Respiratory Infection (SARI) surveillance system, Surveilans Infeksi Saluran Pernafasan Akut Berat Indonesia (SIBI), was established in 2013. While respiratory illnesses such as SARI pose a significant problem, there are limited incidence‐based data on influenza disease burden in Indonesia. This study aimed to estimate the incidence of influenza‐associated SARI in Indonesia during 2013‐2016 at three existing SIBI surveillance sites.

**Methods:**

From May 2013 to April 2016, inpatients from sentinel hospitals in three districts of Indonesia (Gunung Kidul, Balikpapan, Deli Serdang) were screened for SARI. Respiratory specimens were collected from eligible inpatients and screened for influenza viruses. Annual incidence rates were calculated using these SIBI‐enrolled influenza‐positive SARI cases as a numerator, with a denominator catchment population defined through hospital admission survey (HAS) to identify respiratory‐coded admissions by age to hospitals in the sentinel site districts.

**Results:**

From May 2013 to April 2016, there were 1527 SARI cases enrolled, of whom 1392 (91%) had specimens tested and 199 (14%) were influenza‐positive. The overall estimated annual incidence of influenza‐associated SARI ranged from 13 to 19 per 100 000 population. Incidence was highest in children aged 0‐4 years (82‐114 per 100 000 population), followed by children 5‐14 years (22‐36 per 100 000 population).

**Conclusions:**

Incidence rates of influenza‐associated SARI in these districts indicate a substantial burden of influenza hospitalizations in young children in Indonesia. Further studies are needed to examine the influenza burden in other potential risk groups such as pregnant women and the elderly.

## 1 INTRODUCTION

Influenza epidemics are estimated to result in about 3‐5 million cases of severe illness, and 250 000‐500 000 deaths globally each year, with the heaviest burden of disease among certain high‐risk groups (children, elderly, and chronically ill).^1^

Increasing awareness of influenza in Indonesia and other tropical countries has highlighted the need for surveillance to provide timely and high‐quality epidemiology and virology data to enable monitoring of influenza seasonality, identify and monitor high‐risk groups, establish influenza activity baseline levels, facilitate vaccine strain selection, and determine influenza burden to guide policymakers.^2^ While outpatient influenza surveillance for influenza‐like illness (ILI) is widespread, inpatient SARI‐based influenza surveillance has been increasing globally over the past decade, particularly in lower‐income and tropical settings.^3^

**Figure 1:**
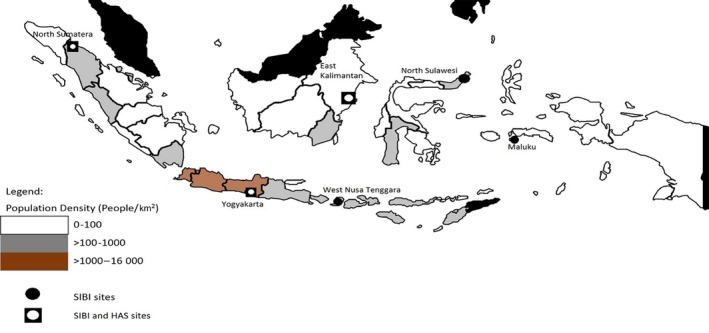
Location of *Surveilans Infeksi Saluran Pernafasan Akut Berat Indonesia* (SIBI) and Hospital Admission Survey sentinel sites

In Indonesia, a SARI surveillance system was established in 2013 through a collaboration between National Institute of Health Research and Development (NIHRD) and the Directorate General of Disease Control and Prevention (Acute Respiratory Infection (ARI) Sub‐Directorate), called Surveilans Infeksi Saluran Pernafasan Akut Berat Indonesia (SIBI). This system provides comprehensive epidemiology and virology data for acute respiratory hospitalizations. There are 6 sentinel hospitals located in 6 different provinces, which also represent geographical areas of Indonesia: Haulussy Hospital in Maluku (east), Bitung Hospital in North Sulawesi (north), Kanudjoso Djati Hospital in East Kalimantan (central), Deli Serdang Hospital in North Sumatra (west), Wonosari Hospital in Yogyakarta (central), and West Nusa Tenggara Provincial Hospital in West Nusa Tenggara (south) (Figure 1).^4^

Driven by the lack of incidence‐based data on influenza‐associated SARI in low‐ and middle‐income countries such as Indonesia, the World Health Organization published a manual in 2015 outlining several methodologies to estimate influenza incidence using surveillance data. The two primary methods described in the manual are community‐based healthcare utilization survey (HUS) and hospital admission survey (HAS). While HUS‐based incidence calculations have been performed to estimate influenza burden in India^5^ and elsewhere, there are limited data available to date on influenza incidence generated through an HAS‐based approach. The objective of this study was to estimate the incidence rate of influenza‐associated SARI in Indonesia during 2013‐2016 by age‐group, using existing SIBI data and a supplemental HAS to estimate sentinel hospital catchment populations.

## 2 METHODS

### 2.1 Surveillance platform

SIBI is comprised of six sentinel facilities at general hospitals serving all age‐groups and socioeconomic levels in six of Indonesia's 34 provinces (Figure 1). Four (Deli Serdang, Wonosari, Bitung, and Kanudjoso Djati) are primary referral district hospitals, and two (Haulussy and West Nusa Tenggara) are secondary referral provincial hospitals. Enrollment of all patients meeting the surveillance case definition is conducted every day year‐round in all inpatient wards except for the obstetric and surgical wards. Surveillance staff use the WHO SARI case definition: hospitalized patients with history of fever or fever ≥38°C, cough, and onset of symptoms within 10 days. Information on demographic characteristics, medical history, clinical presentation, and risk factors is collected.^6^ Respiratory specimens are collected and tested for influenza A and B viruses using real‐time reverse‐transcription polymerase chain reaction (rRT‐PCR) based on CDC rRT‐PCR protocol.^7^ All data collected from the system are recorded in a secure online database.^6^ Of the six SIBI sites, three (Wonosari hospital in Gunung Kidul District, Kanudjoso Djati hospital in Balikpapan District, and Deli Serdang hospital in Deli Serdang District) were selected for the current analysis because of the feasibility of accessing quality data from centralized electronic medical records.

### 2.2 Hospital admission survey (HAS)

Among the three participating districts, a hospital admission survey (HAS) was conducted to estimate the catchment population for each district (Fig. S1). In accordance with WHO guidance,^8^ the catchment population was calculated in a two‐step process. First, the catchment area was defined as subdistricts where ≥80% of SARI cases seeking care at the SIBI hospital reside. Second, the catchment population was the portion of the total population in those subdistricts who sought care at the SIBI hospital as opposed to seeking care at other hospitals in the same areas. Using this approach, we retrospectively reviewed all hospital records based on the admission log to identify all patients with diagnosis of ICD.X J.00‐J.99 from May 2013 to April 2014. We then calculated the age‐group (0‐4, 5‐14, 15‐49, 50‐59, 60‐69, ≥70 years) proportion of respiratory disease admissions at SIBI facilities out of the total respiratory disease admissions at all hospitals in the catchment area. This proportion was applied to the district population (National Statistical Bureau data) to generate an estimated catchment population for the SIBI facility, used as the denominator for incidence calculations. The number of admission by age‐group for ICD.X J.00‐J.99 at the three hospitals is presented in Table S1.

### 2.3 Statistical analysis

Demographic and clinical characteristics of influenza‐positive and influenza‐negative SARI cases were tested for differences using Chi‐squared test. Annual incidence rates and 95% confidence intervals were calculated according to the WHO manual, using catchment population denominators defined from the HAS and numerator defined from all SARI influenza‐positive cases enrolled in the surveillance system, and accounting for variance around the numerator of influenza‐positive SARI cases by age stratum, site, and year. Analyses were conducted in microsoft excel© version 2010 (Microsoft Corporation, Redmond, WA, USA) and spss version 21 (IBM Corporation, Armonk, NY, USA).

### 2.4 Ethical considerations

This study was reviewed and approved by Ethical Clearance Committee of National Institute of Health Research and Development (NIHRD), Ministry of Health of Indonesia (No. LB.02.01/5.2/KE.198/2013 for 2014, LB.02.01/5.2/KE.324/2015 for 2015).

## 3 RESULTS

### 3.1 SARI surveillance findings

**Figure 2:**
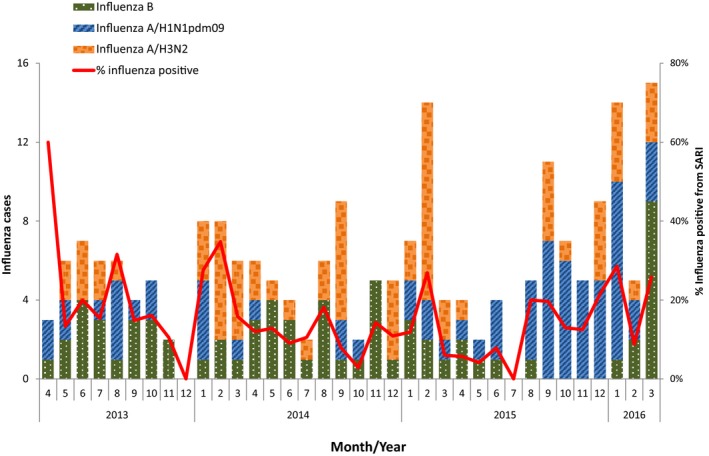
Influenza‐positive severe acute respiratory infection (SARI) cases by month, May 2013‐April 2016

In the 3‐year study period (May 2013 to April 2016), 1527 SARI cases were enrolled in the 3 SIBI facilities: 444 cases at Wonosari hospital, 708 cases at Kanudjoso Djati hospital, and 375 cases at Deli Serdang hospital. The majority (1392 [91%]) of SARI cases provided respiratory specimens of which 199 (14%) were positive for influenza. The remaining 135 (9%) SARI cases refused to have a laboratory specimen collected for influenza testing. Most influenza‐positive SARI cases were children under 5 years (n = 114, 57%) followed by children aged 5‐14 years (n = 58, 29%); only 27 influenza‐positive SARI cases (14%) were aged >14 years (Table 1). This age distribution was similar to that of all 1527 enrolled SARI cases. Asthma (7%), diabetes (3%), and chronic obstructive pulmonary disease (2%) were the most frequent comorbidities reported among influenza cases, and 4% of influenza‐positive cases were current smokers. Only four influenza cases (2%) reported having influenza vaccination in the last 12 months, all were aged ≥50 years old. Oseltamivir was prescribed to less than one percent of admissions, including one influenza case and 6 non‐influenza cases. One (<1%) influenza case died during hospitalization; this was a 61‐year‐old patient who was admitted 1 day after illness onset, diagnosed with renal failure and given antibiotics but did not receive antiviral treatment. This fatal case had a reported influenza vaccination within the previous 12 months. During the study period, cocirculation of multiple influenza virus types and subtypes occurred, with virus detection year‐round, and peaks in January or February each year (Figure 2).

**Table 1:** Demographic and clinical characteristics of severe acute respiratory illness (SARI) cases at three hospitals in Indonesia, May 2013 to April 2016

Variable	Influenza test		P‐value
	Positive (N = 199)	Negative (N = 1193)	
	n (%)	n (%)	
Gender			
Male	107 (54)	645 (54)	.94
Female	92 (46)	548 (46)	
Age‐group			
0‐4 years	114 (57)	797 (67)	.02^a^
5‐14 years	58 (29)	226 (19)	
15‐49 years	12 (6)	91 (8)	
50‐59 years	5 (3)	38 (3)	
60‐69 years	6 (3)	25 (2)	
>70 years	4 (2)	16 (1)	
Medical History			
Asthma	13 (7)	74 (6)	.71
Diabetes	5 (3)	21 (2)	.6
Chronic obstructive pulmonary disease	4 (2)	28 (2)	.95
Cardiovascular disease	2 (1)	8 (0.7)	.53
Tuberculosis	2 (1)	9 (0.8)	.41
Chronic kidney disease	1 (0.5)	5 (0.4)	.91
Hematology disorder	1 (0.5)	3 (0.3)	.82
Current smoker	2 (1)	40 (3)	.67
Prescribed Oseltamivir	1 (0.5)	6 (0.5)	.73
Influenza Vaccination	4 (2)	7 (1)	.08
Outcome (N = 1458)^b^	N = 185	N = 1145	
Recovered	178 (96)	1103 (96)	.29
Died	1 (0.5)	6 (0.5)	
Others (e.g., forced discharged^c^, referred, unknown)	6 (3)	36 (3)	

a Significant, *P*‐value ≤.05.

b Patients with completed discharge forms.

c Left against medical advice.

### 3.2 Estimated catchment population of sentinel sites

For the HAS, we collected inpatient data at 5 hospitals in Gunung Kidul, 9 hospitals in Balikpapan, and 6 hospitals in Deli Serdang including the SIBI hospitals. The overall proportion of those admitted for respiratory illness at SIBI sites out of the total respiratory admissions to all facilities in the catchment area was 52% for Wonosari hospital, 26% for Kanudjoso Djati hospital, and 51% for Deli Serdang hospital, although this proportion varied by age‐group at each site (Table 2). Based on population data from National Statistical Bureau (n = 331 773 in Gunung Kidul, n = 594 322 in Balikpapan, and n = 288 236 in Deli Serdang), the estimated catchment population for each sentinel site was 172 064 in Gunung Kidul, 105 458 in Balikpapan, and 130 110 in Deli Serdang.

**Table 2:** Estimated catchment population by age for three hospitals in Indonesia, May 2013 to April 2016

Age‐group (year)	Wonosari				Kanudjoso Djati				Deli Serdang			
	SARI cases (influenza‐positive cases)	Population	% Population seeking care for respiratory illness at hospital (95% CI)	Estimated catchment population for sentinel site	SARI cases (influenza‐positive cases)	Population	% Population seeking care for respiratory illness at hospital (95% CI)	Estimated catchment population for sentinel site	SARI cases (influenza‐positive cases)	Population	% Population seeking care for respiratory illness at hospital (95% CI)	Estimated catchment population for sentinel site
	A	B	C	D = B × C	A	B	C	D = B × C	A	B	C	D = B × C
0‐4	331 (35)	23 577	59 (52‐67)	13 945	428 (52)	59 673	14 (13‐16)	8542	237 (27)	31 923	56 (51‐60)	17 742
5‐14	96 (21)	50 639	55 (45‐64)	27 664	126 (22)	103 975	11 (9‐13)	11 515	85 (15)	56 609	41 (35‐47)	23 115
15‐49	9 (1)	153 865	54 (46‐62)	82994	68 (3)	348 020	18 (16‐20)	61 664	35 (8)	161 150	42 (38‐47)	68 349
50‐59	4 (2)	44 234	54 (44‐65)	24 083	33 (3)	54 349	31 (27‐36)	12 624	11 (0)	23 295	51 (45‐58)	11 990
60‐69	3 (0)	31 418	44 (35‐54)	13 964	33 (6)	20 458	39 (34‐45)	7902	4 (0)	9845	57 (50‐65)	5642
≥70	1 (0)	28 040	43 (37‐49)	12 062	20 (4)	7847	40 (35‐46)	3211	3 (0)	5414	60 (53‐68)	3272

### 3.3 Annual incidence rate of influenza‐associated SARI

The combined incidence of influenza‐associated SARI varied during 3 years, ranging between 13 and 19 per 100 000 population, with an increase in the point estimate each year (Table 3). Among the three hospitals, the influenza‐associated SARI incidence was highest in children aged 0‐4 years (82‐114 per 100 000 population) followed by those aged 5‐14 years (22‐36 per 100 000 population). Incidence varied significantly across sites (*P* < .05, ANOVA); Kanudjoso Djati was the only hospital that reported influenza‐positive cases in patients aged ≥60 years. At this site, observed incidence among those ≥60 years was higher than that among younger adults (15‐59 years), but lower than that among children aged <15 years.

**Table 3:** Estimated incidence rates per 100 000 population and 95% confidence intervals for influenza‐associated severe acute respiratory illness by year and hospital site, May 2013 to April 2016

Age‐group (year)	Wonosari			Kanudjoso Djati			Deli Serdang			All Three Sites		
	2013‐2014	2014‐2015	2015‐2016	2013‐2014	2014‐2015	2015‐2016	2013‐2014	2014‐2015	2015‐2016	2013‐2014	2014‐2015	2015‐2016
0‐4	71 (56‐90)	85 (69‐106)	92 (75‐113)	280 (249‐315)	175 (151‐203)	152 (129‐178)	5 (2‐12)	33 (24‐47)	113 (94‐136)	87 (70‐136)	82 (66‐101)	114 (95‐137)
5‐14	10 (6‐19)	32 (23‐45)	32 (23‐45)	86 (70‐107)	43 (32‐58)	60 (47‐78)	4 (1‐10)	29 (20‐42)	29 (20‐42)	22 (14‐33)	33 (23‐46)	36 (26‐50)
15‐49	1 (0.2‐7)	0 (0)	0 (0)	0 (0)	3 (1‐9)	1 (0.3‐7)	4 (1‐11)	2 (0.9‐9)	4 (1‐11)	1 (0.4‐7)	1 (0.4‐7)	1 (0.4‐7)
50‐59	0 (0)	4 (1‐10)	4 (1‐10)	8 (3‐ 15)	16 (9‐25)	0 (0)	0 (0)	0 (0)	0 (0)	2 (0.5‐8)	6 (2 ‐13)	2 (0.5‐8)
60‐69	0 (0)	0 (0)	0 (0)	13 (7‐21)	25 (17‐37)	38 (28‐52)	0 (0)	0 (0)	0 (0)	3 (1.3‐10)	7 (3‐15)	11 (6‐20)
≥70	0 (0)	0 (0)	0 (0)	62 (48‐79)	0 (0)	62 (48‐79)	0 (0)	0 (0)	0 (0)	11 (6‐20)	0 (0)	11 (6‐20)
Total	8 (4‐16)	12 (7‐22)	13 (7‐22)	36 (26‐49)	24 (16‐36)	24 (16‐36)	3 (1‐10)	11 (6‐20)	22 (15‐34)	13 (8‐23)	15 (9‐25)	19 (12‐30)

## 4 DISCUSSION

During 2013‐2016, our study identified influenza as an important contributor to respiratory disease hospitalizations in three geographically diverse areas of Indonesia. The greatest impact was among young children aged <5 years, who had the highest incidence across all three sites, followed by older children aged 5‐14 years. This trend persisted in annual site‐specific estimates, despite variability in incidence across sites and between seasons. For one site where incidence rates could be calculated for older age‐groups, a bimodal distribution was observed where incidence rates were highest in young children, low in the productive age‐groups, and then higher in those aged 60 years or more. This is consistent with previously observed patterns of influenza disease burden where the highest risk of complications occurs among young children and older adults.^9^ Importantly, like other studies reporting on influenza infections and incidence in older populations in Indonesia, both the number of influenza cases detected and the associated underlying catchment population are small.^10^, ^11^ This may reflect the population age structure in Indonesia where the majority of the population is young, different health‐seeking behavior for acute respiratory infections among older people or difficulties in enrolling older patients meeting the SARI case definition. Further studies are needed to better understand influenza burden in older age‐groups.

Our estimates of incidence of influenza‐associated SARI, which use a recently published methodology by the WHO, are lower than estimates reported by neighboring countries. Singapore had an age‐specific influenza‐associated hospitalization rate for pneumonia and influenza (based on ICD–coded discharge diagnosis) of 28.3 (95% CI 24.7‐32.7) during 2004‐2008 and 29.6 (95% CI 19.7‐40.6) during 2010‐2012.^12^ Thailand had an incidence of influenza‐associated pneumonia hospitalization across all age‐groups of 83 per 100 000 persons in 2005, 22 per 100 000 persons in 2006, 42 per 100 000 persons in 2007, and 66 per 100 000 persons in 2008.^13^ Further, our estimates for children under 5 years were also lower than what has been reported elsewhere.^14^, ^15^, ^16^, ^17^ Differences in influenza incidence might be explained by the use of different case definitions, calculation methods, variability in influenza burden by year, or differences in care seeking, physician admitting practices between study populations, and possibility of enrollment performance for SARI cases. In addition to that, SARI only represents about a half of all respiratory and a quarter of all respiratory and circulatory hospitalizations attributable to influenza. Substantial differences in influenza burden are not uncommon across different settings, and can be seen both across countries and within the same country. In India, influenza incidence differed between two population‐based platforms conducting inpatient surveillance during the same time period, despite similar methods and same time period of surveillance. In this study, the annual influenza‐associated hospitalization rates were 20.3‐51.6 per 10 000 (in Vadu) and in the other area (Ballabgarh) 4.4‐6.3 per 10 000 population.^5^

These findings also contribute to the broader understanding of influenza circulation patterns, health‐seeking behavior relating to influenza and oseltamivir prescription practices in Indonesia. The cocirculation of different influenza types and subtypes during the 3‐year study period reinforce earlier surveillance findings^11^ that Indonesia has year‐round influenza activity but with yearly peaks in December‐February. This trend is similar to other tropical countries and may become relevant as control measures are introduced during future seasonal influenza outbreaks or during pandemic influenza events.^18^ Eleven (2%) of SARI cases received influenza vaccine in the previous year. Indonesia currently recommends influenza vaccination for pilgrims going to Hajj.^19^ Influenza vaccines are available in the private sector but are not currently subsidized by the Government of Indonesia. The current findings may become useful to guide the timing of vaccine campaigns if other population groups are targeted.

Our analysis is subject to several limitations. Given the small size of our estimated catchment population ≥70 years (n = 18 545 for all three sites), the study was underpowered to calculate incidence of influenza hospitalizations in this age‐group, who are a known risk group for severe disease from influenza.^1^ While the SIBI platform attempts to identify all SARI cases in participating hospitals, there might be some underdetection, which could result in underestimation of influenza burden in these settings. All three hospitals provided care to patients of all ages, but SARI cases were predominately under 5 years old. Routine phone calls, 6‐monthly site visits and twice‐yearly SIBI surveillance system meetings with all hospitals aimed to reduce the potential for biased enrollment of patients, but we cannot rule out differences in enrollment practices by ward or by age‐group. The ability of SIBI to identify cases of influenza is influenced by the sensitivity and specificity of the SARI case definition. It is possible that the SARI case definition is less sensitive to detect influenza among elderly patients.^20^, ^21^ Further studies are needed to further estimate burden in elderly populations in Indonesia. Additionally, for catchment population denominator, we applied the 2013‐2014 estimates as generated from the HAS to calculate yearly incidence rates for all 3 years and therefore did not capture any potential changes in catchment population over time. The direction of the impact of using 2013‐2014 catchment population data to calculate subsequent year incidence rates is unknown.

Care seeking for severe influenza likely varies by age‐group in this population, with young children more likely to visit a hospital and be admitted. Differences in care seeking and public health system performance^22^ may also influence the differences in our findings between the three sites, as incidence was highest in Balikpapan district, compared to two other districts. It is possible because Kanudjoso Djati hospital is the only large and modern hospital in its region, and influenza patients compared to other SARI patients may directly seek care there compared to smaller hospitals. Finally, this study was conducted only at three of the 6 SIBI sentinel sites, and captures less than 50% of the country's population.

## 5 CONCLUSIONS

In this study, the hospitalized influenza burden was highest in young children, consistent with other countries, and in line with their inclusion as a key target group for vaccination as recommended by WHO Strategic Advisory Group of Experts on Immunization (SAGE).^23^ These trends are consistent with what is found elsewhere and underscore the impact of pediatric admissions for influenza on health systems in lower‐income settings. We suggest a further study on cost‐effectiveness of vaccines among children aged 6 months to 5 years to provide information to guide policymakers on influenza vaccination among specific target groups.

## DISCLOSURE

This work was supported by the U.S. Centers for Disease Control and Prevention [Cooperative Agreement Number 5U51IP000346‐05].

## DISCLAIMER

The findings and conclusions in this manuscript are those of the authors and do not necessarily represent the views of the Centers for Disease Control and Prevention.
