# Supplementary material for: Estimated incidence of influenza‐associated severe acute respiratory infections in Indonesia, 2013‐2016
Source: Influenza Other Respir Viruses. 2017 Dec 5;12(1):81–7. doi: 10.1111/irv.12496 (PMC5818340; doi:10.1111/irv.12496)

**Supplemental Table 1.** Number (%) of admissions for J00-J99 for May 2013 - April 2014 by age group

| **Age Group (year)** | **Wonosari** | **Kanudjoso Djati** | **Deli Serdang** |
| --- | --- | --- | --- |
|  |  |  |  |
| 0 – 4 | 164(18%) | 2306(36%) | 538(28%) |
| 5 - 14 | 108(12%) | 1187(18%) | 240(13%) |
| 15 - 49 | 165(18%) | 1946(30%) | 547(29%) |
| 50 - 59 | 90(10%) | 451(7%) | 204(11%) |
| 60 - 69 | 108(12%) | 306(5%) | 171(9%) |
| ≥70 | 265 (29%) | 270 (4%) | 182 (10%) |
| **Total** | **900** | **6466** | **1882** |

**Supplemental Figure 1.** Description of hospital admission survey (HAS) to calculate catchment population.


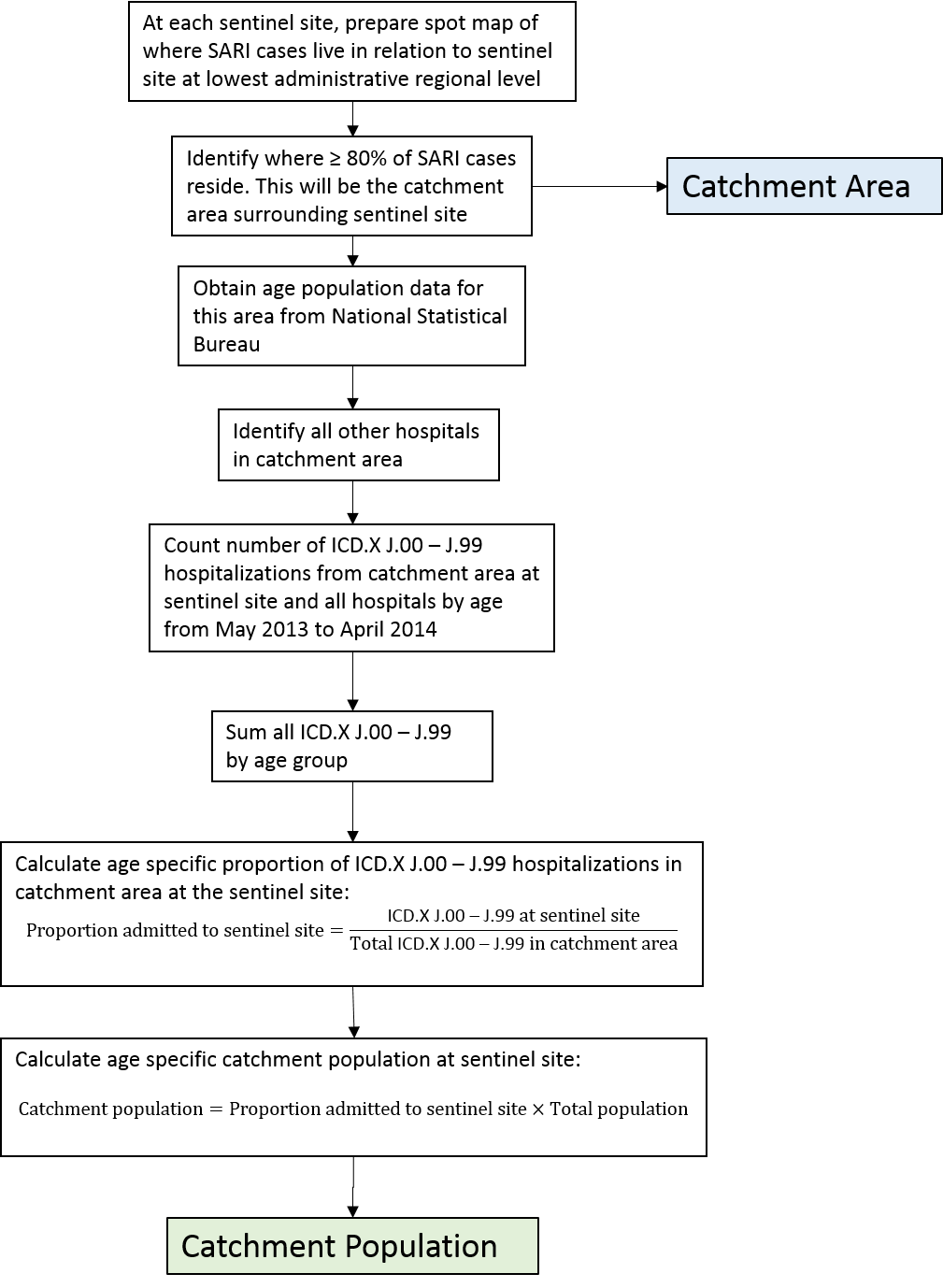

Supplement: Supplementary file 1 [file IRV-12-81-s001.docx]
